# Conformable Device for Independent Measurements of Mucosal and Vascular Barriers in a Complex In Vitro Intestinal Model

**DOI:** 10.1002/advs.202511686

**Published:** 2025-12-21

**Authors:** Alexandra Elisabeth Wheeler, Sonja Blasche, Rob Bradley, Rachana Acharya, Sophie Oldroyd, David Bulmer, Kiran R. Patil, Róisín Meabh Owens

**Affiliations:** ^1^ Department of Chemical Engineering and Biotechnology University of Cambridge UK; ^2^ MRC Toxicology University of Cambridge UK; ^3^ Department of Pharmacology University of Cambridge UK

**Keywords:** Conformable device, multi‐barrier model, multi‐barrier monitoring

## Abstract

Electrochemical approaches for monitoring cell barrier integrity have become important read‐out tools for in vitro models. Conventional commercially available electrochemical devices are however poorly suited for monitoring the more complex and multi‐dimensional models which have recently been developed thanks to advances in tissue engineering. In particular, commercially available devices are typically unable to provide barrier‐by‐barrier resolution in multi‐barrier models. In this study, a gut‐immune‐vasculature model is presented and utilized to investigate the potential for a novel conformable device to independently monitor two cell barriers within the same model‐device platform. The platform, supported by cytokine analysis, is first used to study known bacterial metabolites and dietary compounds, showing changes in epithelial and/or endothelial barrier resistance. The platform is also used to test complex mixtures of bacterial metabolites produced by four intestinal bacteria individually as well as by a community of 25 diverse gut bacteria. The study demonstrates the potential for employing tailored in vitro intestinal models coupled with novel electrochemical device monitoring for host‐microbe interactions studies.

## Graphical abstract

Development of more complex in vitro models necessitates the development of improved monitoring approaches. This work demonstrates a novel conformable device capable of measuring the resistance of two cell barriers within a single model independently, a significant advantage over conventional trans‐epithelial/endothelial electrical resistance (TEER) monitoring platforms which only provide bulk measurements.

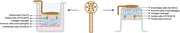

## 1 Introduction

Non‐invasive and label‐free bioelectronic technologies are important for monitoring in vitro biological models [1]. Trans Epithelial Electrical Resistance (TEER) monitoring is one such technique used to monitor in vitro barriers and commonly involves two electrodes placed, in media, on either side of a cell monolayer. Various commercially available TEER monitoring devices have been developed [2] include the Epithelial Volt/Ohm Meter, known as the EVOM (World Precision Instruments) [3] and the automated CellZscope (nanoAnalytics) [4], amongst others. While operation varies between these platforms, the read out in all cases is a measure of the resistance of the cell barrier to ion flow, providing insight into the permeability of a cell monolayer [2, 3, 5].

Advances in tissue engineering have enabled the development of more physiologically relevant in vitro models including 3D and biomimetic approaches which better mimic the in vivo microenvironment [6]. These include the use of 3D matrices, electroactive scaffolds [7], hydrogels [4], organoids, multiple cell types, lab‐on‐chip platforms [8] and 3D‐printing approaches, amongst others [6]. Barrier monitoring for models which incorporate multiple epithelial and/or endothelial monolayers is however challenging and thus far techniques to monitor two barriers in the same model independently are lacking. The commercially available TEER monitoring platforms mentioned above, which are recommended predominantly for single monolayer models, provide a bulk measurement between the electrodes and thus, if two cell monolayers lie between them, the measured resistance value incorporates both. Some barriers, for example endothelial cells, such as human umbilical vein endothelial cells (HUVECs), have a significantly lower monolayer barrier resistance [9, 10] compared to epithelial cell barriers, such as those of the enterocyte cell line Caco2 [11, 12]. Measurements across both using the conventional techniques are unable to provide information regarding each barrier independently with readings dominated by the monolayer of highest resistance, providing little or no information regarding the lower resistance barrier. More recent 3D‐like scaffold approaches [7] have also not yet been shown to provide barrier‐by‐barrier resolution.

Previous work in our group has demonstrated the development of an all‐planar conformable thin‐film bioelectronic device capable of measuring at the air‐liquid‐interface, offering enhanced mechanical flexibility as well as higher sensitivity compared to the conventional barrier monitoring techniques mentioned above [2]. Here, we develop a multi‐barrier model and investigate the ability of an updated version of this device to monitor changes in two barriers independently, within the same tissue culture insert. We present an in vitro gut‐immune‐vasculature model, incorporating both epithelial and endothelial cell barriers separated by an immune cell‐laden collagen hydrogel, and use this to demonstrate the conformable device's ability to measure the resistance of each barrier independently, still in a label‐free and non‐invasive manner. We then validate the model‐device platform's response to known bacterial or dietary compounds/metabolites, namely the short‐chain fatty acid (SCFA) butyrate, and the long‐chain fatty acid palmitic acid; as well as explore the effects of more complex gut bacterial supernatants from individual bacteria and a 25‐strain synthetic community. To demonstrate multi‐read out capabilities of the model we additionally measure changes in cytokine levels resulting from cellular responses to the various metabolites. Development of more complex and immune‐competent models is key to metabolic research [8] and improving our understanding of microbiome‐related dynamics in over‐all health and disease. The enhanced method for monitoring changes within these models, as we present here, represents the next frontier for metabolic and cardio‐metabolic research.

## 2 Results and Discussion

### 2.1 Establishment of a Multi‐Barrier In Vitro Model

**FIGURE 1:**
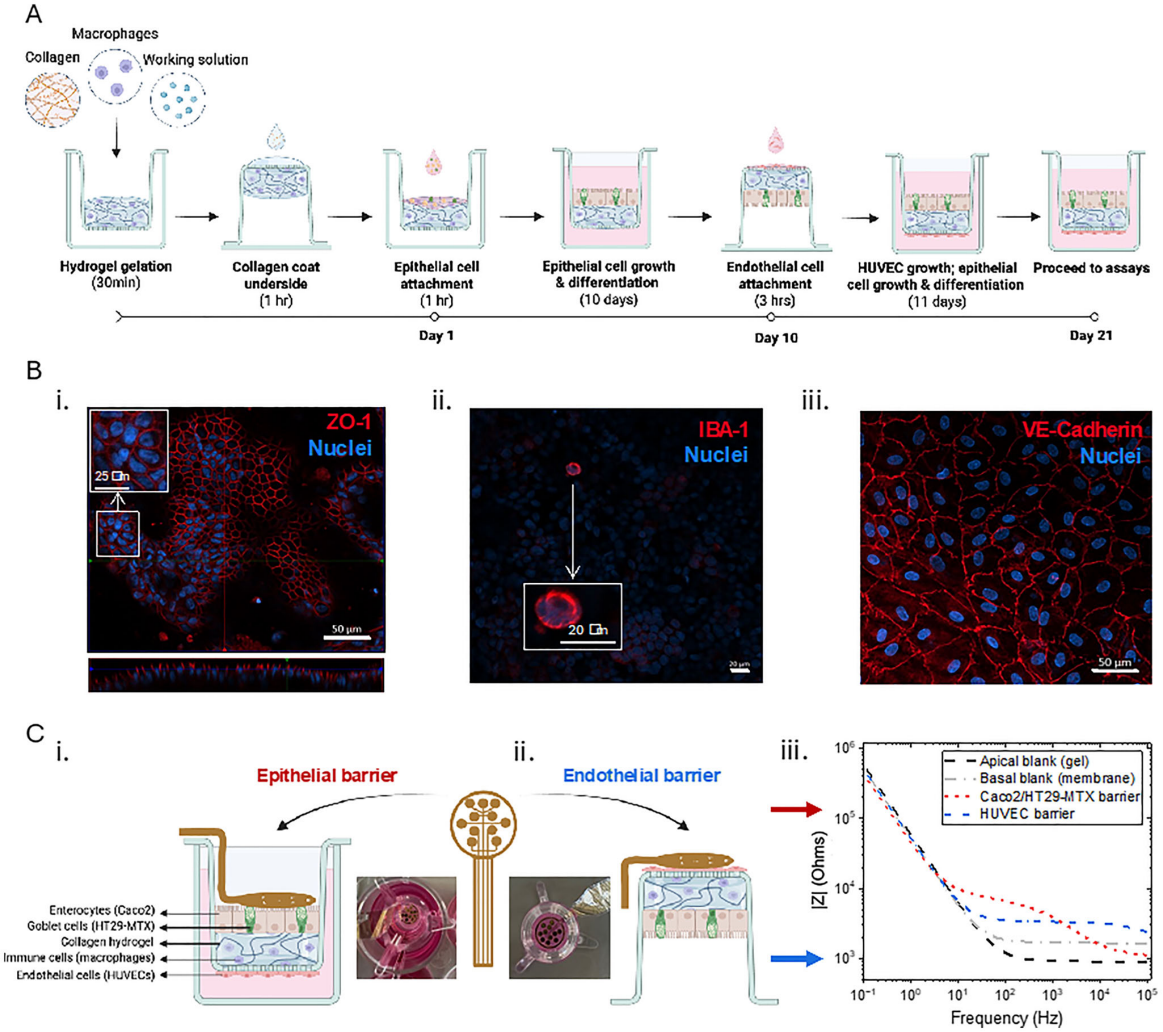
(A) Schematic of the quad‐culture model development. A macrophage (THP‐1 derived) laden hydrogel is first formed inside a tissue culture insert from a mixture of rat tail type I collagen, working solution and macrophages in culture media. Following 30 min of gelation, the inserts are inverted and the underside of the insert membrane is collagen coated. The insert is then inverted again and a 3:1 ratio of Caco2 (enterocytes) and HT29‐MTX (goblet) cells are seeded on top of the hydrogel and allowed to attach. Epithelial cells are allowed to grow and differentiate for an initial period of 10 days. Thereafter, the inserts are inverted and HUVECs are seeded on the underside of the membrane and allowed to attach. The model is then inverted again and grown for a further period of 11 days (21 days total for epithelial barrier) prior to further end point assays being performed. Created with Biorender.com. (B) Optical characterization of the quad‐culture model. (i) Immunofluorescence confocal image of the epithelial cell barrier comprised of Caco2 and HT29‐MTX cells grown on the soft hydrogel surface displaying complex undulating topology. Inset showing an exploded view of a cell area. Cells are stained for ZO‐1 proteins (red) and nuclei (Hoechst; blue). Scale bar: 50 µm. (ii) Immunofluorescence confocal image of a macrophage within the collagen hydrogel, with inset showing an exploded view of the macrophage. Stained for ionized calcium binding adaptor molecule 1 (IBA‐1) protein (red) and nuclei (Hoechst; blue). Scale bars: 20 µm. (iii) Immunofluorescence confocal image of the endothelial cell barrier comprised of HUVEC cells grown on the underside of the cell culture insert membrane. Cells are stained for vascular endothelial cadherin (VE‐cadherin) proteins (red) and nuclei (Hoechst; blue). Scale bar: 50 µm. (C.i, ii) Schematic and photographs of the conformable device and iii. representative impedance spectra. Images and schematics showing the device i. contacting the apical epithelial (Caco2/HT29‐MTX) barrier while the tissue culture insert rests in the well plate; and (ii) contacting the basolateral endothelial (HUVEC) barrier on the underside of the tissue culture insert following insert removal from the well plate and inversion onto a flat surface. Created with Biorender.com. (iii) Ability of the conformable device to independently measure the two cell barriers in the same model showing characteristic impedance Bode plot spectra from measurement of the epithelial (Caco2/HT29‐MTX) barrier and endothelial (HUVEC) barrier. Apical and basal blank spectra differ due to the gel vs membrane interface on the apical and basal side, respectively.

The cell‐culture insert based gut‐immune‐vasculature model incorporates an epithelial cell barrier, comprising a co‐culture of Caco2 (enterocytes) and HT29‐MTX (mucus‐producing [13] goblet‐like) cells, grown within the insert on top of a collagen‐laden hydrogel [4]; with an endothelial HUVEC barrier on the underside of the insert membrane. Establishment of this “quad‐culture” model is multi‐step with the epithelial barrier growing and differentiating over a period of 21 days and the HUVEC monolayer forming over 11 days (Figure 1A).

Following the 21 day model development period, formation of the epithelial and endothelial barriers was visually confirmed using immunofluorescence (Figure 1B). While the endothelial cells, seeded on the rigid insert membrane, formed a planar layer (Figure 1B.iii); growth of the epithelial cells on the softer hydrogel surface resulted in increased surface topology showing an undulating surface (Figure 1B.i), more similar to the in vivo environment. Presence of the immune cells within the central hydrogel was also confirmed (Figure 1B.ii). Viability of all cell types after 21 days was confirmed using a live/dead stain (SI Figure S1B), with both epithelial and endothelial barriers as well as the central immune cells displaying predominantly live cells. To further evaluate barrier formation over the model development period, the gold standard EVOM was also used to monitor TEER three times per week, with stabilization in TEER seen toward the end of the period as expected (SI Figure S1A). Following model establishment, the ability of the novel conformable device to measure the barriers within the model was next considered.

### 2.2 Ability of the Flexible Device to Monitor Epi‐ and/or Endothelial Barriers

The updated device presented here is downsized to make it compatible with 24‐well tissue culture inserts and incorporates 11 equally sized working electrodes (WE) 400 µm in diameter, the designated sensing cutoff size determined previously [2], along with an internal planar counter/reference (CE/RE) electrode. The design and fabrication of this device was as previously described, using standard lithography techniques [2]; and electrochemical impedance spectroscopy (EIS), using a 2‐electrode set‐up with the counter and reference electrodes shorted, was again employed to monitor barrier changes.

The ability of the conformable device to measure both epithelial and endothelial monolayers in isolation was first confirmed on simplified models comprising of a single monolayer of each respectively, grown directly on conventional tissue culture insert membranes. Each barrier displayed distinct EIS curves with both showing a characteristic barrier plateau in the impedance spectra (SI Figure S2A‐B). A previously described four element cell circuit model [2] was used to model the barriers, comprising series and parallel resistance and capacitance elements [R1(R2C2)C1], where the parallel R2 and C2 represent the resistance and capacitance of the cell barrier, respectively [2]. R2 is therefore the metric of interest, representing the resistance of the cell barrier similar to TEER. The ability of the devices to measure changes in both of these barriers independently was also confirmed through the addition of calcium chelating agent ethylene glycol tetraacetic acid (EGTA) to induce transient barrier disruption with subsequent recovery confirmed (SI Figure S2).

Following the above validation studies, the ability of the device to measure the two barriers independently when part of the same multi‐barrier quad‐culture model was next considered. To do this, the device was first carefully placed on one barrier and then the other. Apical measurements were taken while inserts remained in the well plate with apical media removed (Figure 1C.i). In order to contact the endothelial barrier, seeded on the underside of the cell culture insert membrane, inserts were removed from the plates and inverted, with the underside of the membrane facing upward and the epithelial monolayer positioned upside down. The device was then carefully placed on the HUVEC barrier and recordings taken (Figure 1C.ii). Viability of both the epithelial and endothelial cells post measurement was confirmed using a live/dead stain (SI Figure S3). In this model, not only does the hydrogel provide a more biomimetic surface for the epithelial cells to grow, being softer than the rigid non‐physiological membrane and providing an extracellular matrix‐like structure to house immune or other cells; but it also acts as an ion reservoir, crucial for electrical recordings of the HUVEC barrier.

Using this approach, the feasibility of measuring each barrier independently while they form part of the same model was confirmed (Figure 1C.iii), representing an important advantage over conventional TEER monitoring technologies, such as the EVOM^TM^, which only provide a single bulk measurement across both barriers. These conventional bulk measurements are dominated by the stronger epithelial barrier, providing little to no information regarding the weaker HUVEC barrier (SI Figure S1A). With the conformable device however, the weaker endothelial barrier can be clearly distinguished from the stronger barrier formed by the epithelial layer (Figure 1C.iii).

To further study the selectivity of the device in isolating changes in each individual barrier, the quad‐culture model was reproduced as a computational simulation with finite‐element modelling of the applied electric field to evaluate the contribution of individual barrier layers to the overall impedance response of the conformable device. A schematic of the model is presented in SI Figure S4A. The electric potential was mapped as a colour map in two dimensions ranging from 0.01 V which is the applied potential at the working electrode (red end of spectrum) to 8 × 10^−7^ V (blue end of the spectrum). As shown in SI Figure S4B, the blank condition (cell culture medium only, black curve) displayed a low and nearly frequency‐independent impedance, consistent with a purely resistive electrolyte. Introduction of a HUVEC monolayer (blue curve) increased the impedance in the mid‐frequency range, indicating additional capacitive behavior associated with the endothelial barrier. The Caco‐2/HT29‐MTX epithelial co‐culture (red curve) exhibited a further increase in total impedance and a pronounced plateau region at low frequencies, characteristic of a tight, highly resistive barrier with significant polarization effects at the electrode interface. The Bode plots were extracted from the simulation of the electric potential and are a close match to the experimental results.

Finite‐element simulations of the electric potential distribution corroborated these experimental observations (SI Figure S4C). In the blank configuration, the electric field lines extended uniformly through the electrolyte, showing minimal attenuation. The electric potential distribution also indicates a majority of the applied potential being focused near the working electrode surface, as expected. Upon addition of the endothelial layer (HUVEC), the potential drop is localized near the barrier interface, indicating restricted ionic conduction across the layer. Upon applying the device to the epithelial layer (Caco2‐HT29 MTX) on the apical side in the quad‐culture configuration shows a similar distribution of the electric potential across the cell layer and confined the electric field near the electrode surface, confirming that increased barrier tightness effectively impedes current flow and elevates the impedance magnitude observed experimentally. In both cases, the simulation demonstrates that application of the conformable device on either end of the model restricts the electric potential to the nearest cell layer only and will not interfere with the cell layer present on the opposite side of the model. Together, these results demonstrate that the model successfully captures both the frequency‐dependent electrical characteristics and spatial potential distribution associated with each biological layer in an independent and selective manner.

The flexibility of the conformable device demonstrated here therefore presents a new and unique way to directly monitor multiple barriers in a single model independently, offering a new dimension for monitoring more complex models.

### 2.3 Effects of the Short‐Chain Fatty Acid Butyrate

SCFAs, which are the primary metabolites of dietary fiber fermentation by intestinal microbiota, are thought to play numerous roles in modulating gut function and are generally thought to have positive effects along the gut‐immune axis and in overall health [14, 15, 16]. Butyrate in particular has been shown to play an important role in maintaining intestinal barrier function and integrity as well as providing an energy source for colonocytes [14, 17, 18]. It is thought to have anti‐inflammatory effects on monocytes, stimulating the production of anti‐inflammatory interleukin‐10 (IL‐10) [19]. Further, it has been suggested that butyrate plays a positive role in cardiovascular health [20], with studies indicating beneficial effects on endothelial barrier integrity [20, 21]. Here, the effects of butyrate on barrier integrity and cytokine profiles, on both the gut‐ and vasculature sides of the model, was therefore explored.

**FIGURE 2:**
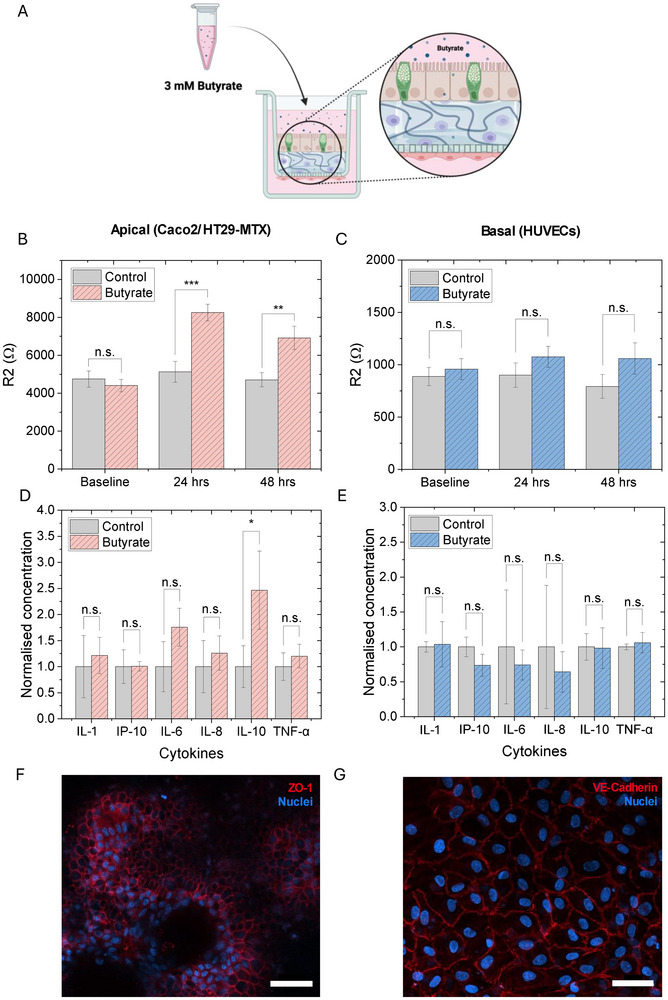
Effects of 3 mM butyrate on the quad‐culture model. (A) Schematic showing apical introduction of 3 mM butyrate. Created with Biorender.com. (B,C) Quad‐culture response to butyrate as measured using the conformable devices. (B) Epithelial (Caco2/HT29‐MTX) and (C) endothelial (HUVEC) barrier resistance changes following 24 and 48 h of 3 mM butyrate incubation compared to untreated control wells. Analyzed by *t*‐test (n.s. *p >* 0.05; *p < 0.05; **p < 0.01; ***p < 0.001). Data from three independent experiments with four inserts each (n = 12). Data presented as mean ± SEM. (D‐E) Cytokine profiles in the (D) apical (epithelial) and (E) basal (endothelial) media following 48 h of 3 mM butyrate incubation. Analyzed by *t*‐test (n.s. *p >* 0.05; *p < 0.05). Data from three independent experiments with one insert each (n = 3). Data presented as mean ± SD. (F,G) Immunofluorescence confocal images of the (F) epithelial (Caco2/HT29‐MTX) and (G) endothelial (HUVEC) cell barriers. Undulating hydrogel surface topology is again visible for the epithelial cells. Cells are stained for nuclei (Hoechst; blue) as well as ZO‐1 or VE‐cadherin proteins (red), respectively. Scale bar: 50 µm.

A previous study showed beneficial effects at 2 mM butyrate on Caco2 barrier integrity, while detrimental effects were observed at 8 mM Butyrate [22]. Butyrate concentration in the human intestine varies considerably among individuals and across intestinal regions and is strongly influenced by both microbial composition and diet [23, 24]. Previous in vitro studies have used and recommend butyrate concentrations ranging from 1 to 8 mM [25, 26]. Further, a gut microbial model community comprising of 25 bacteria produced 3 mM—5 mM butyrate in the supernatant [27]. 1, 2, 3 and 4 mM were therefore tested when added apically for 24 h. Based on this optimization study (SI Figure S5), 3 mM butyrate was added apically (on the epithelial side) for 48 h (Figure 2A). A significant increase in epithelial (Caco2/HT29‐MTX) barrier resistance was noted at both 24 and 48 h compared to butyrate‐free control wells (Figure 2B; SI Figure S6). While the same trend was noted for the endothelial HUVEC barrier, increases did not reach statistical significance (Figure 2C; SI Figure S6). Integrity of both barriers was also confirmed visually post treatment and measurement (Figure 2F,G). This change in resistance was also found to correlate with a significant increase in IL‐10 secretion in apical media following 48 h of butyrate incubation (Figure 2D), in line with previous studies [19]. No significant differences were noted apically or basally for other cytokines (Figure 2D,E).

Butyrate is thought to be almost entirely (95%) taken up by colonocytes [28], with only ∼2% entering circulation [29, 30]. The amount reaching the HUVEC layer is therefore likely to be minimal, however it, together with the IL‐10 secreted into the apical media, may contribute to the increasing trend noted in HUVEC resistance while also explaining why changes did not reach significance either in terms of HUVEC resistance or cytokine profile.

### 2.4 Effects of the Long‐Chain Fatty Acid Palmitic Acid

The pro‐inflammatory [31] long chain fatty acid, palmitic acid, often linked with western diets [32] and metabolic disease [31, 33], was next considered. Previous in vitro studies have shown that palmitic acid has negative effects on intestinal barrier integrity, with Caco2‐based models showing increased permeability [34, 35, 36] and inflammatory responses [34, 36] following palmitic acid treatment, leading to increased cytokine expression including IL‐6, IL‐1β, and tumor necrosis factor‐alpha (TNF‐α) [36]. Additionally, palmitic acid has been shown to activate pro‐inflammatory signalling pathways and stimulate increased expression of IL‐1β, TNF‐α as well as CCL2 and CCL4, amongst others, in macrophages and monocytes [31]. High circulating/plasma palmitic acid levels have also been linked to increased risk of cardiovascular disease [32, 37, 38], with in vitro HUVEC‐based studies associating palmitic acid treatment with endothelial dysfunction related to increased reactive oxygen species generation, oxidative stress and autophagy activity [38, 39]. Increased vascular permeability has additionally been seen in mouse microvascular endothelial cells (MVECs) following sodium palmitate treatment, coupled with increased IL‐1β production [40]. This is in line with findings from human studies where IL‐1β has in particular been associated with cardiovascular risk [41, 42].

**FIGURE 3:**
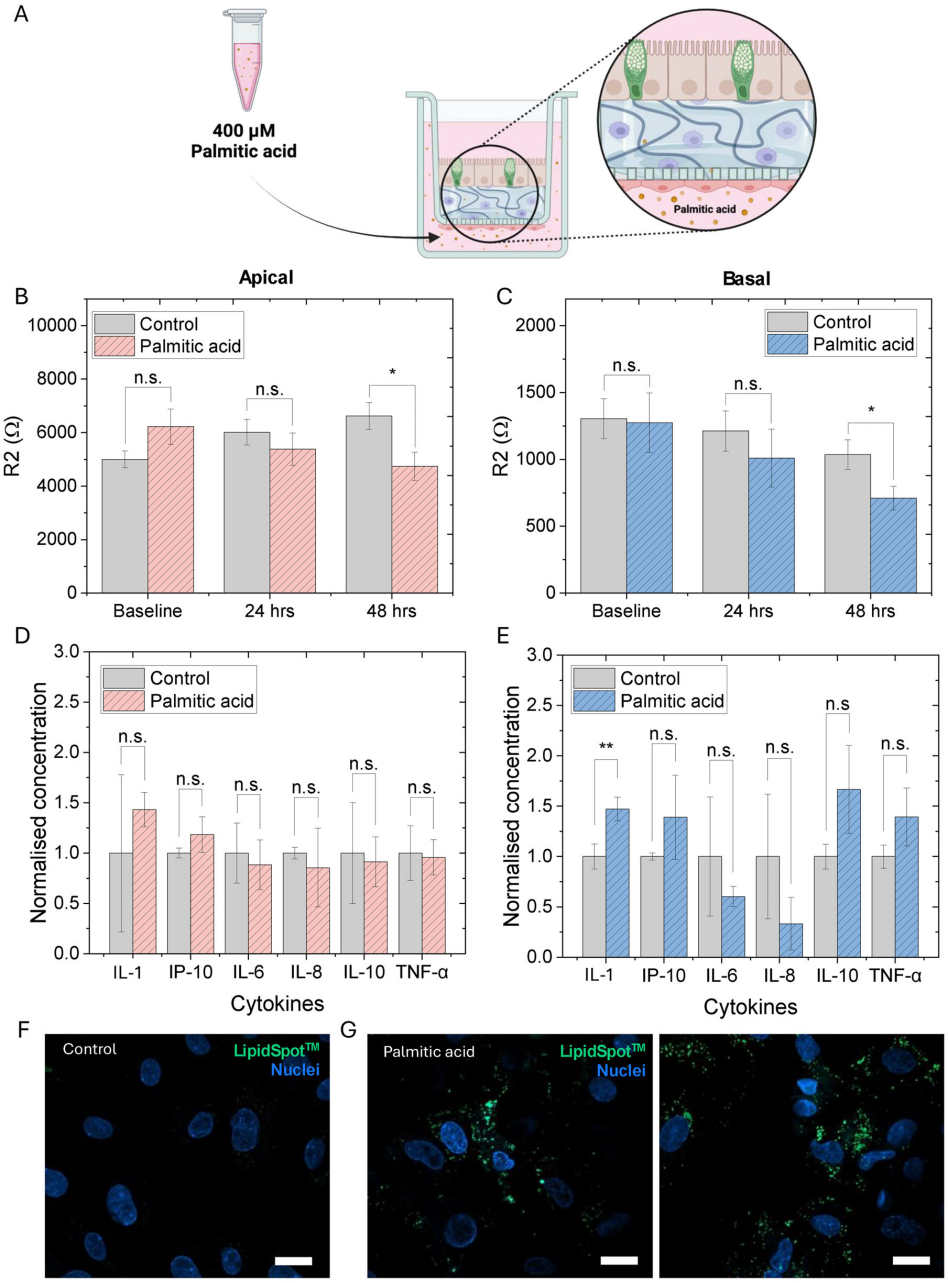
Effect of 400 µM palmitic acid on the quad‐culture model. (A) Schematic showing basal introduction of 400 µM palmitic acid. Created with Biorender.com. (B,C) Quad‐culture response to palmitic acid as measured using the conformable devices. (B) Epithelial (Caco2/HT29‐MTX) and (C) endothelial (HUVEC) barrier resistance changes following 24 and 48 h of 400 µM palmitic acid incubation compared to DMSO/BSA containing control wells. Analyzed by *t*‐test (n.s. *p* > 0.05; *p < 0.05). Data from three independent experiments with three to four inserts each (n = 11). Data presented as mean ± SEM. (D‐E) Cytokine profiles in the (D) apical (epithelial) and (E) basal (endothelial) media following 48 h of 400 µM palmitic acid incubation. Analyzed by *t*‐test (n.s. *p >* 0.05). Data from three independent experiments with one insert each (n = 3). Data presented as mean ± SD. (F,G) Confocal images of the (F) control HUVEC cells with BSA/DMSO only and (G) following palmitic acid incubation. Cells are stained for nuclei (Hoechst; blue) and LipidSpot (green), respectively. Scale bar: 20 µm.

A previous study showed Caco2 cell viability was maintained at palmitic acid concentrations of between 100 and 400 µM, but a significant decrease was noted at 500 µM and above. The same study additionally observed a significant reduction in Caco2 TEER following incubation with 400 µM palmitic acid [36]. To confirm this in the more complex model 300 to 500 µM (added basally), falling within the physiologically relevant plasma free fatty acid concentration (0.2–2 mM) [35], were tested. Based on the aforementioned and an optimization study (SI Figure S7), palmitic acid at 400 µM was introduced to the basal (HUVEC) side of the model (Figure 3A) with changes in epithelial and endothelial barriers monitored over 48 h. A significant decrease in epithelial (Caco2/HT29‐MTX) (Figure 3B; SI Figure S8) and endothelial (HUVEC) (Figure 3C; SI Figure S8) barrier resistance was noted after 48 h compared to the control wells, with increased lipid accumulation in the HUVEC layer after 48 h confirmed visually using a LipidSpot stain (Figure 3F,G). This correlated with increased IL‐1β levels in the basal media of palmitic acid treated cells (Figure 3D), while no significant differences in cytokine levels were noted apically (Figure 3E). As mentioned above, increases in IL‐1β levels have previously been seen in Caco2 cells [36], macrophages [31] as well as endothelial cells [40] following palmitic acid treatment. Further, IL‐1β has previously been shown to affect endothelial and Caco2 tight junctions and lead to an increase in permeability [40, 43, 44] which likely explains the barrier effects observed in this study. The aforementioned demonstrated the ability of the model‐device platform presented here to confirm findings in a single model, which has in previous studies required multiple independent models and precluded real‐time multi‐cell crosstalk.

### 2.5 Effects of Complex Bacterial Supernatants

Butyrate and palmitic acid, being well‐studied compounds with available information relating to the expected effects on the cell lines individually, were used to confirm the responsiveness of the model and the ability of the conformable devices to monitor changes. In isolation however, they represent a simplification and do not capture the complexity of the metabolite profiles produced by food digestion and intestinal bacteria. Therefore, the effects of more complex supernatants from intestinal bacteria were next considered. To this end, bacteria‐free supernatants from four individual gut bacterial strains, as well as from a synthetic community comprising of 25 different bacteria, were considered (**SI** Table S1 **; SI** Table S2, SI Figure S9).

The individual bacteria from which supernatants were generated, namely, *Segatella copri* (*S. copri*)*, Roseburia intestinalis* (*R. intestinalis*)*, Parabacteroides merdae* (*P. merdae*) and *Bacteroides caccae* (*B. caccae*) are predominantly thought to be symbiotic or commensal bacteria, involved in carbohydrate/polysaccharide and amino acid metabolism and SCFA production, amongst others; although their role within the gut can vary [45, 46, 47, 48] (SI Tables S1 and S2). *R. intestinalis* and *P. merdae* are in particular known for their SCFA production, with the former identified as one of the primary butyrate producers in the colon [46, 49] and the latter as a producer of propionate and acetate as well as butyrate to a lesser extent [50]. They have therefore been linked with inflammation prevention, regulation of barrier and immune function, cytokine secretion and shown anti‐atherosclerogenic properties [50, 51], amongst others [46].

Bacteria are however affected by the ecological niches in which they are found, which in the gut is influenced by diet, antibiotics, endogenous factors and other microbes [46]. Study of bacteria and their metabolite profile in isolation may therefore not be representative of their activity when grown together with other individual microbes or in more complex communities [52, 53]. The synthetic community therefore attempts to provide a more complex environment for bacterial growth and metabolite production and comprised of both commensal bacteria and potential pathogens. The latter includes *Clostridioides difficile*, commonly known as *C. diff*, known to cause gastrointestinal disease [54] including diarrhea and colitis (colonic inflammation) [55, 56], while commensal bacteria within the mix included SCFA producing bacteria, such as *R. intestinalis*, introduced above, as well as *Akkermansia muciniphila* [57, 58] and Bifidobacterium species which are thought to mediate intestinal barrier function and immune development as well as reduce inflammation [59, 60].

**FIGURE 4:**
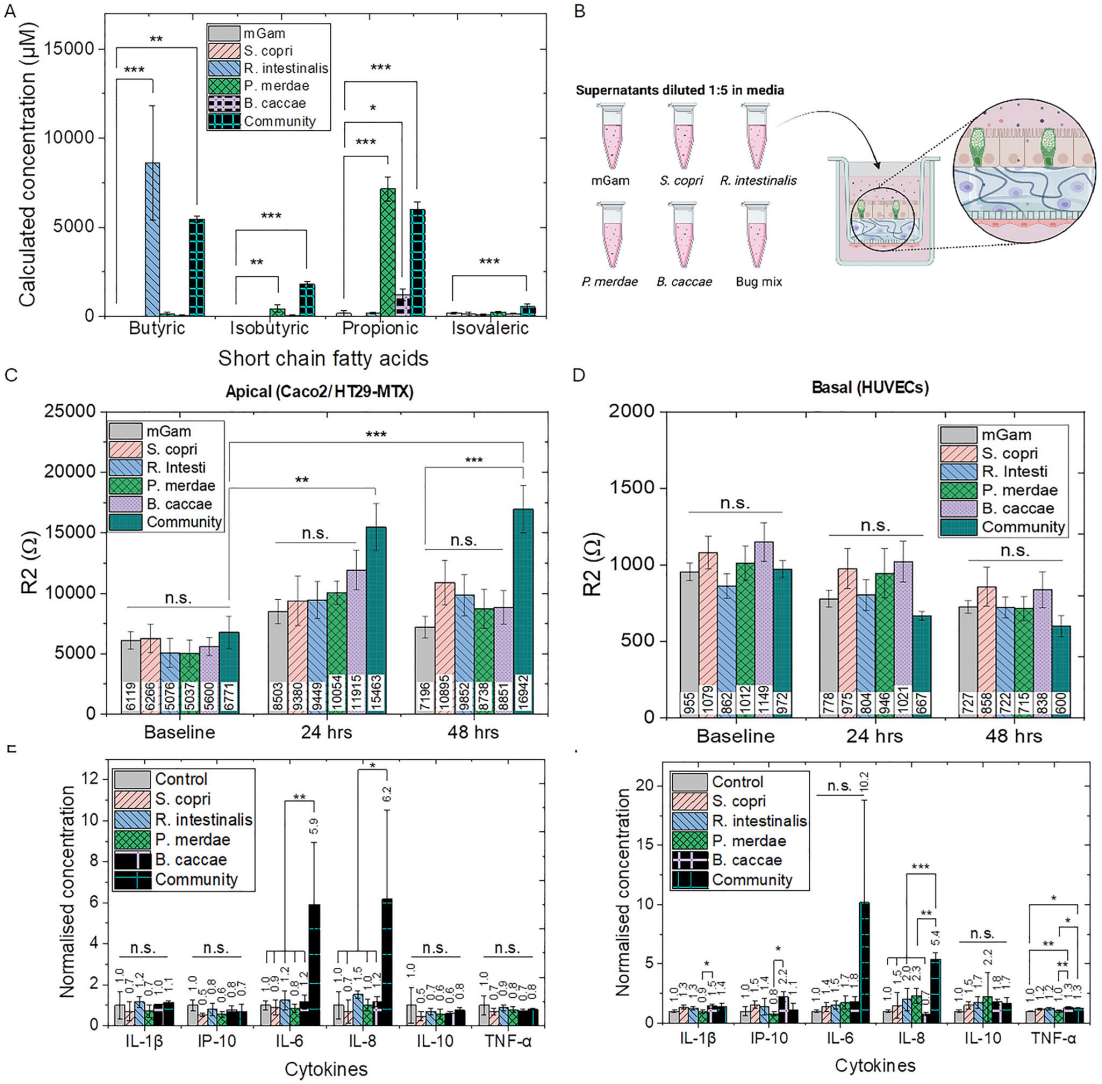
(A) Bacterial supernatant SCFA profiles. Concentration profile showing levels of four SCFAs, namely butyric, isobutyric, propionic and isovaleric acid, in the supernatants from individual bacteria and the synthetic community compared to mGam media (n = 3). Analyzed by one‐way ANOVA, followed by Tukey's post‐hoc test (n.s. *p >* 0.05; *p < 0.05; **p < 0.01; ***p < 0.001). Data presented as mean ± SD. (B–F) Effect of bacterial supernatants on quad‐culture barrier integrity and cytokine profile. (B) Schematic showing apical introduction of mGam bacterial growth media (control) and supernatants from four individual bacteria and the synthetic community. Created with Biorender.com. (C,D) Quad‐culture response to bacterial supernatants as measured using the conformable devices. (C) Epithelial (Caco2/HT29‐MTX) and (D) endothelial (HUVEC) barrier resistance changes following 24 and 48 h of incubation with bacterial supernatants compared to mGam containing control wells. Analyzed by one‐way ANOVA, followed by Tukey's post‐hoc test (n.s. *p >* 0.05; **p < 0.01; ***p < 0.001). Data from three independent experiments with three to four inserts each (mGam n = 12; individual bacteria and synthetic community n = 9 (0 & 48 h)). Data presented as mean ± SEM. (E‐F) Effect of bacterial supernatants on quad‐culture cytokine profiles. Cytokine profiles in the (E) apical (epithelial) and (F) basal (endothelial) media following 48 h of bacterial supernatant incubation compared to mGam control. Analyzed by one‐way ANOVA, followed by Tukey's post‐hoc test (n.s. *p >* 0.05; *p < 0.05; **p < 0.01; ***p < 0.001). Data from three independent experiments with one insert each (n = 3). Data presented as mean ± SD.

Unsurprisingly, based on the above, metabolomics analysis of the supernatants generated from the growth of the abovementioned bacteria revealed significantly different SCFA profiles across the individual bacteria and the synthetic community. As expected, considerable levels of butyric acid, the protonated form of butyrate, were noted in the supernatants generated from the growth of *R. intestinalis* and the synthetic community; while propionic acid, the protonated form of propionate, was in particular noted in the *P. merdae* and synthetic community supernatants. Significant levels of isobutyric and isovaleric acid levels were additionally noted in the synthetic community supernatant (Figure 4A, SI Figure S10). As such, varying, but significant, levels of all four of the SCFAs tested for were found in the supernatant from the synthetic community growth, while significant levels of only one or two were noted in supernatants from individual bacteria, highlighting the increased metabolite complexity generated by multi‐bacteria growth.

These supernatants were then tested, diluted 1:5 in cell culture media, on the quad‐culture model, with effects compared to those of diluted mGam (Figure 4B). While increasing trends were noted in epithelial barrier resistance (R2) following 24 and/or 48 h of incubation with supernatants from the four individual bacteria, changes failed to reach statistical significance. A significant increase in barrier resistance (R2) was however noted following incubation with the synthetic community supernatant (Figure 4C; SI Figure S11; SI Figure S12). Following 24 h of incubation with the supernatant, barrier resistance significantly increased compared to the pre‐supernatant levels however not compared to 24 h of mGam (growth medium) incubation. After 48 h, the increase was significant compared to both pre‐incubation values as well as mGam controls (Figure 4C).

While considerable butyric acid was, as expected, noted in the supernatant from *R. intestinalis*, the concentration once diluted in cell culture media appears too low to individually have a significant effect on barrier resistance. This is in alignment with isolated sodium butyrate tested above which failed to show significant barrier effects below 2 mM (SI Figure S5), with the same likely to be true for the propionic acid produced by *P. merdae*. The complex mixture provided by the synthetic community, which contained measurable levels of all four SCFAs tested however positively influenced barrier integrity (Figure 4C) likely due to the cumulative levels across SCFAs. This highlights the benefit of integrating metabolite profiling with electrical monitoring, as the SCFA data reveals how microbial community composition translates into distinct metabolic outputs and cell barrier responses to provide deeper insight into host–microbe interactions (Figure 4A–D). In future, a lower dilution/higher concentration of the supernatants, guided by in vivo data, in cell culture media should be considered.

No significant differences were noted in the endothelial barrier resistance following incubation with supernatants from either the individual bacteria or the synthetic community compared to the mGam controls (Figure 4D; SI Figure S11; SI Figure S12), in line with the butyrate study above.

While no differences in endothelial barrier resistance were noted, the cytokine profile in the basal endothelial media was more varied than in the apical epithelial media (Figure 4E,F). Apically, significantly higher levels of both IL‐6 and IL‐8 were noted following incubation with the synthetic community supernatant compared to the media controls and other supernatants (Figure 4E). This could be due to the presence of lipopolysaccharides, other metabolites produce by the bacteria and/or presence of the opportunistic pathogens within the mix.

Basally, increased levels of IL‐8 and TNF‐α were noted following incubation of individual bacteria and/or synthetic community supernatants (Figure 4F). This could be due to macrophage activation, cross‐talk from the apical epithelial cells and macrophages and their release of IL‐6 and IL‐8, as well as the other factors mentioned above. Further metabolomic analysis would be useful to identify other metabolites produced by the individual bacteria and the synthetic community which may affect immune activation.

It should be noted that bacterial supernatants represent a complex and incompletely characterized mixture of compounds. Some metabolites are susceptible to oxidation, and some are volatile; both these classes will be affected under experimental design used. Yet not all bacterial metabolites are oxidation prone, and key metabolites implicated in microbe‐host interactions such as SCFA are relatively stable, which we verified by repeat measurements of the same sample over several hours (SI Figure S13). The future experimental set‐ups could be modified to account for possible changes in other metabolites through, for example, designing a closed system connecting bacterial growth medium with the cell culture set‐up.

The findings throughout this study highlight the benefits of the quad‐culture model in allowing cross‐epithelial‐immune‐endothelial signalling in real‐time within the same model. This study also, for the first time, demonstrates the feasibility of independently monitoring changes in both epithelial and endothelial barrier resistance within a single model using a novel conformable device, highlighting the significant opportunity this device presents compared to conventional commercial TEER monitoring platforms. The device‐model combination opens a new paradigm for studying gut‐immune‐vasculature dynamics with significant promise for future studies relating to how diet, microbiota and different metabolites affect intestinal and cardiometabolic health and their roles in metabolic diseases.

## 3 Conclusions

In this study, we have developed a multi‐barrier gut‐immune‐vasculature model and utilized it to test the ability of a novel conformable device to measure changes in epithelial and endothelial barriers independently, a feat not currently possible with conventional TEER monitoring technologies. Improved models of the gut‐immune‐vasculature axis coupled with enhanced non‐invasive monitoring approaches is key to advancing our understanding of metabolic and cardiometabolic diseases, and the role the gut and microbiome play in their development and progression as well as the impact of diet. This study for the first time demonstrates a device capable of monitoring two barriers independently and shows the model to be responsive to bacterial metabolites, both in isolation and when part of more complex supernatants derived from single‐ and multi‐strain bacterial growth. The device‐model platform represents an important advancement for monitoring of multi‐barrier models with significant promise for not only future gut and metabolism‐related studies, but also for modelling and monitoring other tissues in the body.

## 4 Materials and Methods

### 4.1 Cell Culture

Human colorectal adenocarcinoma cell lines Caco2 (RRID:CVCL_0025; male derived) and HT29‐MTX‐E‐12 (HT29‐MTX; RRID:CVCL_G356; female derived) (P53‐59) (ECACC) were routinely cultured in 25 or 75 cm^2^ flasks in complete epithelial growth media comprised of Advanced Dulbecco's Modified Eagle Medium (ADMEM; Gibco) supplemented with 10% Foetal Bovine Serum (FBS; Sigma‐Aldrich), 1% L‐Glutamine (Glutamax‐1; Gibco), 1% penicillin‐streptomycin (Gibco) and 0.1% Gentamicin (Gibco). At cell confluency of 80–90%, cells were passaged using Dulbecco's phosphate‐buffered saline (DPBS) (Sigma‐Aldrich) and 0.25% or 0.05% Trypsin‐EDTA (Gibco), respectively. Monocyte THP‐1 cells (RRID:CVCL_0006), kindly donated by Dr. Graham Christie's laboratory (originally purchased from ECACC), were routinely cultured in suspension in 25 cm^2^ flasks using complete THP‐1 media comprising RPMI 1640 (Gibco) supplemented with 10% FBS (Sigma‐Aldrich), 1% L‐Glutamine (Glutamax‐1; Gibco) and 1% penicillin‐streptomycin (Gibco). HUVECs (Lonza; cryopreserved from pooled donors) (P20‐24) were routinely cultured in 75 cm^2^ flasks in complete endothelial growth media comprised of M199 base media (prepared as described previously [61] through 1:10 dilution of 10× Medium 199, Earle's Salts Medium (M199; Gibco) with sterile water and supplemented with 20% FBS (Sigma‐Aldrich), 1% L‐Glutamine (Glutamax‐1; Gibco), 1.5% 1 M HEPES, 1.8% of 7% (w/v) NaHCO_3_ and 1% penicillin‐streptomycin (Gibco), as well as with growth factors from the EGM‐2 Endothelial Cell Growth Medium BulletKit (Lonza) including hydrocortisone, human fibroblastic growth factor (hFGF), vascular endothelial growth factor (VEGF), R3‐insulin‐like growth factor 1 (R3‐IGF) ascorbic acid, human epidermal growth factor (hEGF), Gentamicin sulfate‐Amphotericin (GA‐1000) and Heparin. At cell confluency of ∼80%, HUVECs were passaged using DPBS (Sigma‐Aldrich) and 0.25% Trypsin‐EDTA (Gibco). Media was refreshed for Caco2, HT29‐MTX and HUVEC cells every 2–3 days and at least once a week for THP‐1 cells. All cells were maintained under humidified conditions at 37°C and 5% CO_2_. Cells were checked regularly to ensure absence of contamination and mycoplasma testing was carried out on each batch received.

### 4.2 Macrophage Preparation

THP‐1 derived macrophages were prepared as described previously [4]. Briefly, to obtain macrophages, monocytes (THP‐1) were differentiated into a macrophage‐like phenotype in 25 cm^2^ flasks through the addition of phorbol 12‐myristate 13‐acetate (PMA) in complete THP‐1 media for 48 h [4], during which time the cells become adherent to the cell culture plastic. Following the 48 h, macrophages were harvested, after two washes with DPBS, using 0.05% Trypsin‐EDTA (Gibco) before being resuspended in complete THP‐1 media (as above). Cells were maintained under humidified conditions at 37°C and 5% CO_2_.

### 4.3 Caco2/HT29‐MTX Co‐Culture Monolayer Growth in Cell Culture Inserts

Caco2 (20 000 cells cm^−2^) and HT29‐MTX (22 000 cells cm^−2^) cells were seeded in co‐culture (3:1 ratio) on top of 24 well ThinCert inserts (Transparent PET membrane, 0.33 cm^2^; Greiner Bio‐One) and maintained in complete epithelial growth media listed above. Cells were cultured for 21 days to allow for cell monolayer formation and differentiation. Media was changed three times a week. Cells were maintained under humidified conditions at 37°C and 5% CO_2_.

### 4.4 HUVEC Monolayer Growth in Cell Culture Inserts

24‐well ThinCert inserts (Transparent PET membrane, 0.33 cm^2^; Greiner Bio‐One) were apically collagen coated, with 50 µL collagen solution, prepared with 100 µL 10% acetic acid, 10 µL 3 mg mL^−1^ collagen (Gibco) and 9.89 mL sterile water, for 1 h. Following washing with DPBS, HUVECs were seeded at 150 000 cells cm^−2^ in the inserts and cultured for 11 days to allow for cell monolayer formation. Media was changed three times a week. Cells were maintained under humidified conditions at 37°C and 5% CO_2_.

### 4.5 Device Fabrication

Devices were micro‐fabricated as described previously [2] using standard photolithography techniques. Briefly, fabrication first involved deposition of an insulating Parylene C layer (2 µm) on a silicon wafer. Photolithography and lift‐off techniques then defined the gold electrode outline, which was subsequently deposited. Thereafter a poly(3,4‐ethylenedioxythiophene)‐poly(styrenesulfonate) (PEDOT:PSS) layer was spin‐coated and cured, reducing impedance and enhancing conductivity. Exposure of the electrode area was facilitated by a sacrificial Parylene C layer. Following fabrication, devices were bonded to a flexible cable. The device comprised of 11 circular working electrodes (WE) all 400 µm in diameter, the designated sensing cut‐off size determined previously [2], along with an internal planar ring counter/reference (CE/RE) electrode.

### 4.6 Conformable Device Recording for Single Barrier Models

A custom PCB was used to connect the device to the external potentiostat (Palmsens 4) to perform impedance measurements, using electrochemical impedance spectroscopy (EIS), from 0.1 – 10^5^ Hz. For each well, readings were taken from at least 3 different working electrodes. To place the electrode on the epithelial/endothelial barrier seeded on top of the cell culture insert membrane, relevant complete growth media was used to fill the well, the device was then carefully placed in the well before all apical media was removed and the device resting fully in contact with the cell monolayer. Readings were then taken for multiple electrodes. To remove the device, media was replaced and the device gently lifted.

### 4.7 Calcium Switch Assay

Epi‐ and endothelial barriers were transiently disrupted using the calcium chelating agent Ethylene Glycol Tetraacetic Acid (EGTA, Alfa Aesar, ThermoFisher, USA). EGTA was dissolved in each complete media at a concentration of 5 mM. Initial baseline conformable device EIS measurements (as described above) were first taken for the cells, where after cells were incubated with the EGTA mixture in the apical and basal compartment for 30 min. EIS measurements were then re‐taken, where after the cells were gently washed with fresh complete media without EGTA, and media replenished. Barrier recovery measurements were taken following overnight recovery. Cells were maintained under humidified conditions at 37°C and 5% CO_2_.

### 4.8 Gut‐Immune‐Vasculature Model Development

Macrophage‐laden collagen hydrogels were formed using rat tail type I collagen (reconstituted at 20 mg mL^−1^ in 0.1% acetic acid). To form 3 mg mL^−1^ collagen gels, a syringe containing chilled stock collagen was attached to a three‐way stopcock luer system together with a chilled working solution comprising 1× PBS, 10× PBS, and 1 M NaOH and mixed. One syringe was then replaced with a third syringe containing macrophages, prepared as above, suspended in complete THP‐1 media and mixed (final concentration of 120 000 macrophages per mL). The combined macrophage‐collagen solution was immediately injected into 24‐well ThinCert inserts (Greiner Bio‐One) and incubated for 30 min under humidified conditions at 37°C and 5% CO_2_ until the gels set and turned opaque. Inserts were then inverted and the underside of the membrane was collagen coated with 50 µL collagen solution, prepared with 100 µL 10% acetic acid, 10 µL 3 mg mL^−1^ collagen (Gibco) and 9.89 mL sterile water, for 1 h at 37°C and 5% CO_2_. The plate was then inverted again and the basal compartment washed three times with DPBS. Caco2 and HT29‐MTX cells were split as indicated above and seeded in a 3:1 ratio on top of the gels with complete epithelial growth media in the apical and basal compartment. The gut‐immune model was maintained in complete epithelial growth media (as above) for an initial period of 10 days to allow for growth and differentiation. Media was refreshed every 2–3 days. On Day 10, HUVECs were split as indicated above. Media was removed from the gut‐immune model plate and the inserts inverted. HUVECs were then seeded at 150 000 cells cm^−2^ on the underside of the inverted cell culture membranes and allowed to attach for 2–3 h. Thereafter, the plate was inverted again and complete epithelial growth media added apically and complete endothelial growth media added basally. The now gut‐immune‐vasculature model was grown for a further 11 days (21 days in total for the Caco2/HT29‐MTX monolayer). Media was changed three times a week throughout. Cells were maintained under humidified conditions at 37°C and 5% CO_2_.

### 4.9 TEER Recording Using Conventional Gold Standard EVOM

TEER measurements were taken using the Epithelial Volt/Ohm Meter (EVOM; World Precision Instruments) with STX2‐Plus electrodes. Prior to recordings, cells were removed from the incubator and allowed to reach room temperature. The electrode height was carefully adjusted to ensure electrodes were submerged in media but not in contact with the cell monolayer, given that the gel raises the height of the epithelial monolayer compared to growth on the insert membrane. TEER recordings taken by the EVOM are in Ohms (Ω) these values were multiplied by the Thincert growth area (0.33 cm^2^) yielding results as Ω.cm^2^ where TEER = (Measured value – blank value) * growth area; averaged per well.

### 4.10 Cell Viability (Live/Dead) Assay

Cell viability was tested using a live and dead cell assay (LIVE/DEAD Viability/Cytotoxicity Kit for mammalian cells, Invitrogen; live: calcein‐AM; dead: ethidium homodimer‐1), following 3 weeks of model culture. The viability of all four cells types (Caco2/HT29‐MTX, macrophages, HUVECs) was checked. The reagents were prepared as per the manufacture's guidance and, after gentle washing of the cells with DPBS, added to both the apical and basal compartments and incubated for 40 min. Thereafter, cells were again washed with DPBS and immediately imaged on a glass‐bottom microscopy dish using an inverted Zeiss LSM800 confocal microscope, using a 10/20× objective lens.

### 4.11 Conformable Device Recording for Multi‐Barrier Model

A custom PCB was used to connect the device to the external potentiostat (Palmsens 4) to perform EIS measurements from 0.1 – 10^5^ Hz. For each well, readings were taken from at least three different working electrodes. To place the electrode on the apical (epithelial) barrier, complete epithelial growth media was used to fill the well, the device was then carefully placed in the well before all apical media was removed and the device was fully in contact with the cell monolayer. Readings were then taken for multiple electrodes. To remove the device, media was replaced and the device gently lifted. For basal (HUVEC) readings, apical media was removed and the insert carefully removed from the plate and inverted using a tweezer. The inverted insert was then gently placed on a clean surface and the device carefully placed on the endothelial cell layer. Readings were then taken for multiple electrodes. To remove the device, a few drop of complete endothelial media were added onto the device and the device gently lifted off. The inserts were then placed back into the well plate and apical media replaced.

For the butyrate, palmitic acid and bacterial supernatant studies detailed below, conformable device measurements were performed at the ALI as described above, with measurements first taken for the epithelial barrier and then the endothelial barrier, during which time the apical media was kept in a separate 24‐well plate. The spectra were modelled with a series resistor‐capacitor (RC) unit followed by a parallel RC unit.

### 4.12 Finite‐Element Simulations

Finite‐element simulations were performed in COMSOL Multiphysics (v6.3) using the Electric Currents physics interface. A 2D cross‐sectional model of the quad‐culture system was constructed, representing the working and counter/reference electrodes separated by electrolyte and hydrogel domains. The electrodes on the conformable device were modelled as 100 nm gold layers with a boundary condition applied for the PEDOT:PSS layer to model it as a surface impedance. These boundary conditions assign electrical properties defined by conductivity σ [S/m], permittivity εr and thickness ds [m] to a surface. This approach was able to reduce the number of elements of the grid while accurately depicting the conformable device in operation. In the quad‐culture model, the matrix laden hydrogel modelled as a layer with finite thickness of 3 mm while the HUVEC and Caco2‐HT29 MTX barriers were treated as boundary conditions with surface impedances. The material properties were defined based on experimentally measured or literature‐reported values: the culture medium (σ = 1.5 S m^−1^, εr = 80), matrix laden hydrogel (σ = 0.05 S m^−1^, εr = 100). The epithelial and endothelial monolayers were assigned electrical properties based on values extracted from the TEER measurements.

A small‐signal frequency domain study was used to simulate the voltage and current response across frequencies from 0.1 Hz to 100 kHz. The potential distribution was visualized at a representative frequency of 100 kHz to highlight the electric potential confinement across different barrier configurations. The simulated impedance spectra were extracted from the ratio of applied potential to electrode current and compared with experimental measurements.

### 4.13 Butyrate Treatment

Butyrate treatment was performed on the quad‐culture model following 21 days of model development, as above. On the day of the experiment, sodium butyrate (Sigma‐Aldrich) was dissolved in complete epithelial growth media (as above) at the required concentration (1–4 mM). This mixture was vortexed immediately before 260 µL was added to the apical compartment of the tissue culture inserts containing the quad‐culture models. Fresh complete endothelial growth media (as above) was added to the basal compartment of the tissue culture well plate. The set‐up was incubated for 24 or 48 h in standard cell culture conditions (5% CO_2_, 37°C). For initial experiments to select the optimal butyrate concentration 1, 2, 3, and 4 mM butyrate were tested with TEER measurements taken using the EVOM^TM^ (as detailed above) following 24 h. In a separate set of experiments, EIS measurements were taken using the conformable device following 24 and 48 h of incubation with the selected 3 mM butyrate. Measurements were taken immediately prior to Butyrate addition as well as after 24 and 48 h of incubation. After the 48 h timepoint, the apical and basal media were collected and immediately frozen at −80°C, for later cytokine analysis.

### 4.14 Palmitic Acid Treatment

As palmitic acid is hydrophobic and poorly soluble in cell culture media, similar to other studies [35, 36, 62], it was first dissolved in DMSO and conjugated with BSA prior to further dilution in cell culture media. To this end, palmitic acid (powder, Sigma‐Aldrich) was dissolved in dimethyl sulfoxide (DMSO) and stored at −20°C. On the day of the experiment, the palmitic acid solution was conjugated with bovine serum albumin (BSA, Fisher) in complete endothelial growth media (as above) before being diluted to the required concentration (300–500 µM). This mixture was vortexed immediately before 840 µL was added to the basal compartment of the quad‐culture model well plate. Fresh complete epithelial growth media (as above) was added to the apical compartment of the tissue culture inserts. The set‐up was incubated for 48 h in standard cell culture conditions (5% CO_2_, 37°C). For experiments to select the optimal palmitic acid concentration, 300, 400 and 500 µM were tested with TEER measurements taken using the EVOM^TM^ (as detailed above) following 24 and 48 h. In a separate set of experiments, EIS measurements were taken using the conformable device following 24 and 48 h of incubation with the selected 400 µM palmitic acid. Measurements were taken immediately prior to palmitic acid addition as well as after 24 and 48 h of incubation. After the 48 h timepoint, the apical and basal media were collected and immediately frozen at −80°C, for later cytokine analysis.

### 4.15 Preparation of Bacterial Supernatants

Culturing was carried out in a vinyl anaerobic chamber (COY) at 37°C with 12% carbon dioxide, 86% nitrogen and 2% hydrogen in mGAM (modified Gifu anaerobic medium broth, HyServe). For monoculture supernatants, bacteria were grown for 24 h, centrifuged for 15 min at 4000 g, the supernatant was collected and sterile filtered (Millex PVDF syringe filter, pore size 0.22 µm, Merck). The microbial community was assembled from 25 bacterial strains, each grown for two passages (each between 1 and 2 days depending on the strain) prior to mixing based on equal OD_600_ units (passage 0). The mixed microbes were then diluted 1:100 into fresh mGAM medium and grown over night (passage 1) and the 1:100 dilution was repeated until passage 4, where the supernatant was harvested by spinning for 15 min at 4000 g and subsequent sterile filtration. Supernatants were stored at −80°C.

### 4.16 Bacterial Supernatant Treatment

Bacterial supernatant treatment was performed on the quad‐culture model following 21 days of model development, as above. On the day of the experiment, bacterial supernatants were diluted 1:5 in complete epithelial growth media (as above). This mixture was vortexed immediately before 260 µL was added to the apical compartment of the tissue culture inserts housing the quad‐culture models. Fresh complete endothelial growth media (as above) was added to the basal compartment of the tissue culture well plate. The set‐up was incubated for 48 h in standard cell culture conditions (5% CO_2_, 37°C). EIS measurements were taken using the conformable device following 24 and 48 h of incubation. Measurements were taken immediately prior to bacterial supernatant addition as well as after 24 and 48 h of incubation. After the 48 h timepoint, the apical and basal media were collected and immediately frozen at −80°C, for later cytokine analysis.

### 4.17 Lactate Dehydrogenase Assay

Cell viability was tested using the CyQUANT lactate dehydrogenase (LDH) Cytotoxicity Assay Kit (Thermofisher, UK), following 3 weeks of model culture and 48 h of treatment either with butyrate or palmitic acid as above. Per the manufacturer's instructions, culture media was incubated with Reaction Mixture for 30 min protected from light, where after the Stop solution was added. Absorbance at 490 and 680 nm was then immediately measured using a plate reader (CLARIOstar^Plus^ BMG Labtech). Cell viability [%] was calculated by comparing treated cells to untreated cells (100%).

### 4.18 Immunofluorescence

Samples were fixed at room temperature in 4% paraformaldehyde (PFA, ThermoFischer Scientific) apically and basally for 7 min, washed 3× with DPBS and stored in DPBS at 4°C. In preparation for immunostaining, samples were incubated with permeabilization solution (DPBS supplemented with 0.1% v/v Triton X‐100 (Fisher)) for 10 min, washed with DPBS and subsequently incubated with blocking solution (DPBS supplemented with 1% (Caco2/HT29‐MTX) or 3% (HUVECS) BSA (Fisher) and 0.1% Tween‐20 (Fisher)) at room temperature for 1 h to prevent nonspecific binding. Cells were then incubated overnight at 4°C with primary antibody, Caco2/HT29‐MTX cells were stained with tight junction protein ZO‐1 (rabbit monoclonal anti‐ZO1; abcam, ab221547; in 1% blocking solution); macrophages with IBA‐1 (abcam) and HUVECs with VE‐Cadherin (Rabbit monoclonal, VE‐Cadherin (D87F2) XP, Cell Signaling Technology; in 2% BSA solution), all diluted in blocking solution. The next day, the samples were washed three times using DPBS and incubated with secondary antibody (goat anti‐rabbit Alexa Fluor 488 (Invitrogen)) diluted 1:400 in blocking solution, for 1 h at room temperature protected from light. Following washing with DPBS, nuclei were counterstained using Hoechst (2 µg mL^−1^ in DPBS) for 10 min. Following thorough washing, samples were stored in DPBS and then imaged on glass‐bottom microscopy dishes using an inverted Zeiss LSM800 confocal microscope, using 10–40× objective lenses. To image the gut‐immune‐vasculature model, HUVECs were first imaged, where after gels were carefully removed from the ThinCert inserts and gently placed upside down on a glass‐bottom microscopy dish for Caco2/HT29‐MTX imaging.

### 4.19 Cytokine Analysis

Cytokine levels were determined using a 5‐Plex Luminex assay (ProcartaPlex: IL‐1β, IL‐6, IL‐8 (CXCL8), IL‐10, IP‐10 and TNF‐α; Life Technologies Limited, Thermo Fisher Scientific, UK). Frozen apical and basal media samples were defrosted on ice. All samples were centrifuges at 10 000 g for 15 min. The samples were then prepared and analyzed following the manufacturers protocol and using the MAGPIX machine.

### 4.20 Metabolomics

Bacterial supernatants were prepared for mass spectrometry by mixing 24 µL of the supernatant with 36 µL of extraction buffer (1:1 methanol: acetonitrile with 0.1% formic acid and internal standards (100×)). Thereafter, the mixtures were vortexed and incubated for 2 h at 4°C before being centrifuged for 5 min at 16 000 g and 4°C. Supernatants were then collected and placed into liquid chromatography–mass spectrometry (LC–MS) vials and stored at −80°C until measurement. Metabolomics analysis of the prepared bacterial supernatants tested for four SCFA's namely butyric acid, isobutyric acid, propionic acid, and isovaleric acid. Caffeine, ibuprofen, donepezil, amoxicillin, closantel, imazalil and acetamiprid, all at 20 µM, were used as internal standards. Analysis was performed on an Agilent 1290 Infinity II LC system coupled with an Agilent 6470 triple quadrupole mass spectrometer with JetStream ESI source operated in dynamic multiple reaction monitoring (dMRM) mode. The SCFAs were measured using a porous graphitic carbon column as described previously [63]. Briefly, 1 µL of sample was injected and analytes were separated on a Thermo Scientific Hypercarb PGC column (3 µm, 50 mm × 2.1 mm) maintained at 40°C. The gradient used a constant flow rate of 0.15 mL min^−1^ and proceeded as follows: 0 min: 100% buffer A (water, 0.1% formic acid) and 0% buffer B (acetonitrile, 0.1% formic acid), 4 min: 60% B, 4.1 min: 100% B, 6 min: 100% B, 6.01 min: 0% B, 9 min: stop time. A pooled QC sample, blanks and serial dilutions of mixed analytical standards were injected regularly throughout each run. Data were normalized to amoxicillin and analyzed using MassHunter Workstation Quantitative Analysis for QQQ v10.1. Concentrations were obtained via external calibration using standard curves.

### 4.21 Statistical Analysis

Data was analyzed using OriginPro, Version 2023b/2025 (OriginLab Corporation, Northampton, MA). Replicate (n) number and statistical tests performed for each experiment are stated in figure captions. In general, statistical comparisons between multiple groups were analyzed using one‐way ANOVA followed by Tukey's post hoc test. Statistical comparisons between two groups were analyzed using an unpaired *t*‐test. A *p*‐value of less than 0.05 was considered statistically significant for all analyses (***p < 0.001, **p < 0.01, *p < 0.05, ns: *p >* 0.05).

## Author Contributions

A.W. and R.M.O. conceptualized the project and the experiments. A.W. performed all model set up, including cell culture, model development and maintenance, as well as butyrate and palmitic acid preparation, bacterial supernatant dilution and all interaction studies and electrical recordings; and performed all confocal imaging and cytokine analysis. S.B. grew the individual bacteria and synthetic community and prepared all bacterial supernatants. S.B. and R.B. performed metabolomics (SCFA) analysis of the bacterial supernatants. S.O. designed and fabricated the flexible devices and assisted with early electrical readings. R.A. performed all finite‐element simulations and associated analysis and plotting. A.W. performed all other data plotting, data analysis, biostatistics, and arrangement of the figures, as well as created the schematics for each figure. The first draft and last draft were written and checked by A.W., and edited by all the co‐authors. The project was supervised by R.M.O., K.P., and D.B.

## Funding

Cambridge Commonwealth, European & International Trust Newnham College UKRI Medical Research Council (Grant nr. MC_UU_00025/11) European Research Council (ModEM, Grant nr. 866028) Air Force Office of Scientific Research (FA8655‐20‐1‐7021).

## Conflicts of Interest

The authors declare no conflicts of interest.

## Supporting information




**Supporting File**: advs73410‐sup‐0001‐SuppMat.docx.

## Data Availability

The data that support the findings of this study are openly available in CAM at https://doi.org/10.17863/CAM.119215, reference number 119215.
