# Supplementary material for: Conformable Device for Independent Measurements of Mucosal and Vascular Barriers in a Complex In Vitro Intestinal Model
Source: Adv Sci (Weinh). 2025 Dec 21;13(13):e11686. doi: 10.1002/advs.202511686 (PMC12955888; doi:10.1002/advs.202511686)
Supplement: Supplementary file 1 — Supporting File: advs73410‐sup‐0001‐SuppMat.docx. [file ADVS-13-e11686-s001.docx]

**Supporting Information: Conformable device for independent measurements of mucosal and vascular barriers in a complex *in vitro* intestinal model**

*A. Wheeler^1^, S. Blasche^2^, R. Bradley^2^, R Acharya^1^, S. Oldroyd^1^, K. R. Patil^2^, D. Bulmer^3^, Róisín M. Owens^1^*

^1^Department of Chemical Engineering and Biotechnology, University of Cambridge

^2^MRC Toxicology, University of Cambridge

^3^Department of Pharmacology, University of Cambridge

**SI Fig.S1** shows TEER readings using the EVOM™ over the quad-culture model set up period, providing bulk measurements across the model. TEER associated with HUVEC monolayers is expected to be significantly lower^[1,2]^ than epithelial barriers such Caco2^[3,4]^ and Caco2/HT29-MTX co-cultures, bulk measurements are therefore expected to be dominated by the epithelial barrier. To confirm this, TEER of a HUVEC-only monolayer was compared to the quad-culture model and a large disparity noted, clearly showing that EVOM™ measurements are unlikely to provide any information regarding development or changes in the HUVEC barrier, with measurements dominated by the upper epithelial barrier.

**SI Fig.S1. A.** Quad-culture vs HUVEC-only TEER as measured using the gold-standard EVOM^TM^. TEER results taken using the EVOM^TM^ for the dual-barrier quad-culture model over the 21 day model set up period (red) and for an endothelial (HUVEC) monolayer alone (blue). Data from two independent experiments with three inserts each (n = 6). Data presented as mean $\pm$ SEM. Schematic showing EVOM^TM^ recording of the quad-culture model or HUVECs only (right). Model schematics created with Biorender.com. **B.** Confocal images of the Live/Dead viability assay showing survival of epithelial (left; Scale bar: 100 $\mu$m), immune (centre; Scale bar: 50 $\mu$m) and endothelial (right; Scale bar: 100 $\mu$m) cells following 21 days of model set up. Live cells: green; Dead cells: red. (Live/Dead Viability/Cytotoxicity Kit for mammalian cells, Invitrogen; live: calcein-AM; dead: ethidium homodimer-1)

**SI Fig.S2** demonstrates the ability of the conformable devices to measure both Caco2/HT29-MTX and HUVEC barriers independently as well as their ability to measure barrier changes due to the addition of calcium chelating agent ethylene glycol tetraacetic acid (EGTA) which induces transient barrier disruption. Following 30 minutes of EGTA incubation, the spectra for both monolayers flattened and approached the insert baseline (**SI Fig.2A-B**), with extracted R2 values confirming significant loss of barrier function (**SI Fig.2C-D**). Recovery of the barriers was confirmed following overnight incubation with fresh calcium-containing media, with the characteristic impendence spectra plateaus returning (**SI Fig.2A-B**) and extracted R2 values showing no significant differences compared to pre-treatment baselines (**SI Fig.2C-D**).

**SI Fig.S2.** Electrical characterisation of Caco2/HT29-MTX and HUVEC-only monolayers using the conformable device. (**A-B**) Representative EIS Bode plot curves from **A.** the Caco2/HT29-MTX co-culture monolayer model and **B.** the HUVEC-only monolayer model, showing the insert baseline (black), the cell barriers (red), barrier disruption following 30 minutes of incubation with EGTA (blue) and following overnight recovery in fresh calcium containing media (green). **(C-D)** Normalised R2 values as measured using the conformable device for **C.** the Caco2/HT29-MTX co-culture monolayer model and **D.** HUVEC-only model: pre-incubation with EGTA, following 30 minutes incubation with EGTA, and following overnight recovery in fresh media. Data from three inserts each (n = 3). Analysed by one-way ANOVA, followed by Tukey’s post-hoc test (n.s. p>0.05; **p<0.01; ***p<0.001). Data presented as mean ± SD. Insets created with Biorender.com. Circuit schematic inset in **C** shows the four-element equivalent circuit used to model the cell barriers; where R1 represents the combined resistance of the device, insert and basal media; C1 the capacitance of the device and; R2/C2 in parallel the resistance and capacitance of the cell barrier respectively.

**SI Fig.S3** shows cell viability following measurements with the flexible device.

**SI Fig.S3.** Cell viability post measurement. Confocal images of the Live/Dead (Calcein/viability assay showing survival of epithelial (left), immune (centre) and endothelial (right) cells following measurements with the flexible device. Live cells: green; Dead cells: red. Scale bar: 50 $\mu$m. (Live/Dead Viability/Cytotoxicity Kit for mammalian cells, Invitrogen; live: calcein-AM; dead: ethidium homodimer-1)

**SI Fig. S4.** shows finite-element analysis that quantifies how electrode geometry governs field spread and layer selectivity. Using a 2D Electric Currents model of the full quad-culture stack, mapping and parameter sweeps of the working-electrode was performed on each contact side (apical vs basal). The simulations show that the drive field concentrates beneath the working electrode and across the nearest cell interface. These results clarify how selecting the contact side and frequency band enables the device to isolate changes in individual barrier signals, consistent with the measured apical/basal datasets.

| A  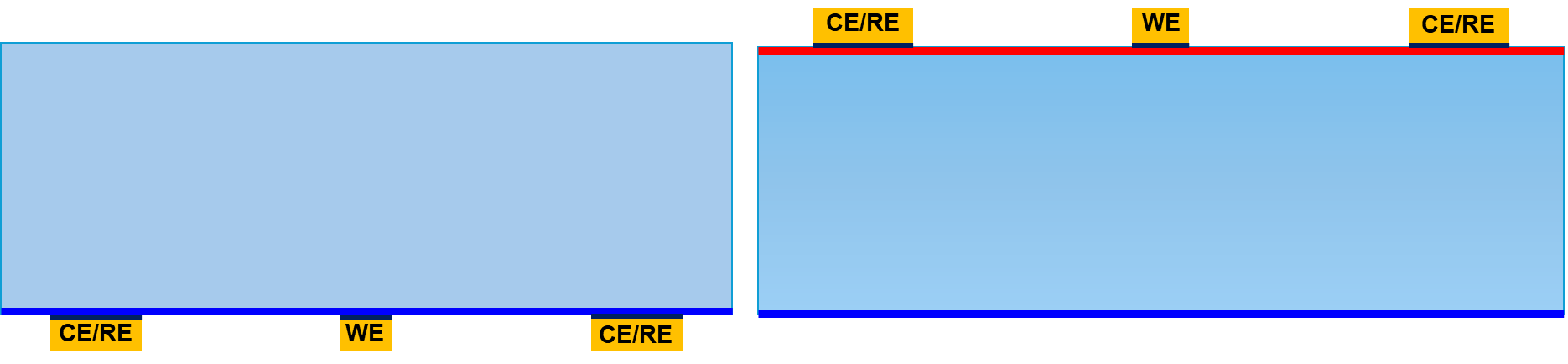  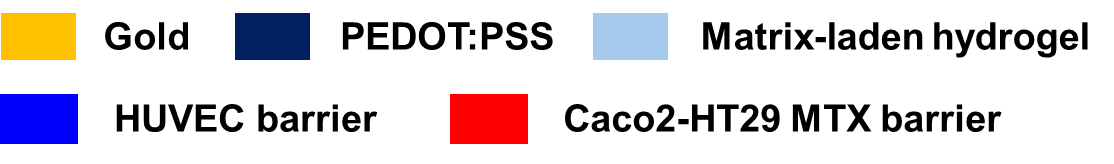  B   | C  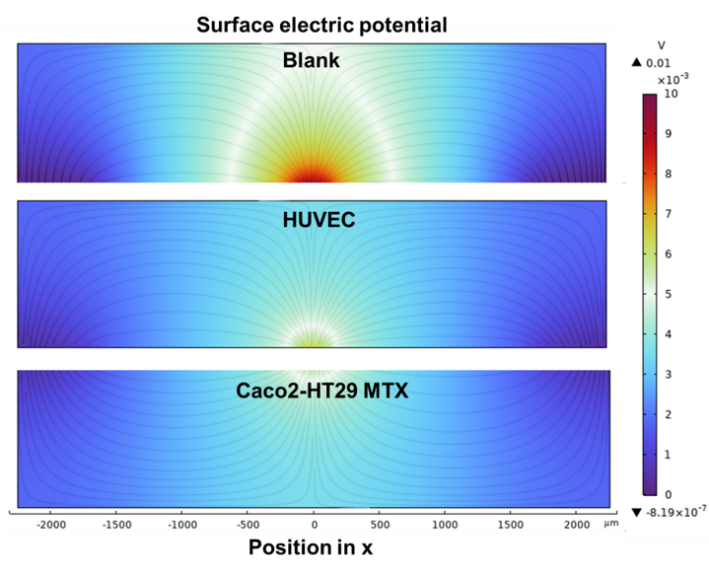 |
| --- | --- |

**SI Fig.S4. A** Schematic visualization of the quad-culture model geometry as applied in the COMSOL simulation model (not to scale for emphasis). **B.** Extracted bode plots from a simulation of the quad-culture model demonstrating the variation of impedance magnitude |Z| as a function of frequency for three conditions: the blank electrolyte, device placed on the HUVEC barrier and device placed on the Caco2-HT29 MTX barrier. **C.** Visualization of the surface electric potential applied during operation for a blank condition (electrolyte only), device applied on the HUVEC layer and the device applied on the Caco2/HT29-MTX layer in the quad-culture model.

**SI Fig.S5** shows the results of an optimisation study using the gold standard EVOM™ to test different concentrations of butyrate on the model as a whole (**Fig.S5A)**. A previous study showed beneficial effects at 2 mM butyrate on Caco2 barrier integrity, while detrimental effects were observed at 8 mM Butyrate^[5]^. We therefore initially tested 1, 2, 3 and 4 mM added apically for 24 hours, with 3 mM found to have the most significant positive effect on measured TEER (**Fig.S5B**). Cell viability was also confirmed at this concentration using a lactate dehydrogenase (LDH) assay (**Fig.S5C-D**). No significant changes in cell viability were noted apically for the epithelial cells (**Fig.S5C**) or basally for the endothelial cells (**Fig.S5D**) following treatment with 3 mM butyrate apically.

**SI Fig.S5.** Butyrate concentration optimisation. **A.** Schematic showing introduction of butyrate apically. Created with Biorender.com. **B.** Effects of 1–4 mM butyrate added apically on barrier integrity as measured by the gold standard EVOM^TM^, compared to control wells with no butyrate. TEER values following 24 hours of butyrate incubation. Analysed by one-way ANOVA, followed by Tukey’s post-hoc test (n.s. p>0.05; *p<0.05; **p<0.01; ***p<0.001). Data from four independent experiments with one to three inserts each (n control = 9; n 1 mM = 5; n 2 mM = 6; n 3 mM = 6; n 4 mM = 5). Data presented as mean ± SD. (**C-D**) Lactate dehydrogenase (LDH) results from **C.** apical and **D.** basal media indicating epithelial and endothelial viability is maintained following treatment with 3 mM butyrate (added apically) compared to control wells to which no butyrate was added. Analysed by t-test (n.s. p>0.05). Data from two independent experiments with three inserts each (n = 6). Data presented as mean ± SD.

**SI Fig.S6** shows representative EIS data in Bode and Nyquist plot format for epithelial (Caco2/HT29-MTX) and endothelial (HUVEC) barriers following apical incubation with butyrate.

**SI Fig.S6.** Butyrate treatment: Impedance spectra for the quad-culture model showing gel (apical) or membrane (basal) baselines, cell barrier baselines and cell barriers following 24 and 48 hours of butyrate treatment in (**A-B**) Bode plot format and (**C-D**) in Nyquist plot format. Plots showing raw data (solid lines) and fitted data (dotted lines) obtained using the resistor (R) and capacitor (C) circuit [R1(R2C2)C1]where R2 is the barrier resistance and metric of interest. (**A** **and** **C**) showing apical epithelial (Caco2/HT29-MTX) barrier changes (0 hrs - R2: 4717 Ω, R2 error: 10.93 %; 24 hrs – R2: 7889 Ω; R2 error: 9.77%; 48 hrs – R2: 8092 Ω; R2 error: 13.57%) and (**B and D**) showing basal endothelial barrier changes (0 hrs - R2: 1473 Ω, R2 error: 35.44%; 24 hrs – R2: 1599 Ω; R2 error: 23.39 %; 48 hrs – R2: 1885 Ω; R2 error: 26.33%).

**SI Fig.S7** shows the results of an optimisation study using the gold standard EVOM™ to test different concentrations of palmitic acid on the model as a whole (**SI** **Fig.S7A**). A previous study showed Caco2 cell viability was maintained at palmitic acid concentrations of between 100 and 400 $\mu$M, but a significant decrease was noted at 500 $\mu$M and above. The same study additionally observed a significant reduction in Caco2 TEER following incubation with 400 $\mu$M palmitic acid^[6]^. To confirm this in the more complex model 300 to 500 $\mu$M (added basally), falling within the physiologically relevant plasma free fatty acid concentration (0.2–2 mM)^[7]^, were tested. In alignment with the previous study, a significant decrease in TEER was observed following 400 $\mu$M treatment (**SI** **Fig.S7B**). Cell viability was additionally confirmed at this concentration using the LDH assay, which showed no significant differences apically (**SI** **Fig.S7C**) or basally (**SI** **Fig.S7D**). Based on the aforementioned, and the results of the previous study^[6]^, the 400 $\mu$M concentration, added basally, was selected for further studies using the conformable device.

**SI Fig.S7.** Palmitic acid concentration optimisation. **A.** Schematic showing introduction of palmitic acid basally. Created with Biorender.com. **B.** Effects of 300 – 500 $\mu$M palmitic acid (dissolved/conjugated with DMSO/BSA), added basally, on epithelial barrier integrity as measured by the gold standard EVOM^TM^, compared to control wells with either DMSO/BSA or nothing added. TEER values following 48 hours of incubation. Analysed by t-test (n.s. p>0.05; *p<0.05). Data from two independent experiments with three to four inserts each (n = 7 except 300 $\mu$M n = 6). Data presented as mean ± SD. (**C-D**) Lactate dehydrogenase (LDH) results from **C.** apical and **D.** basal media indicate epithelial and endothelial viability is maintained following treatment with 400 and 500 $\mu$M palmitic acid (added basally), compared to control wells with either DMSO/BSA or nothing added. Analysed by t-test (n.s. p>0.05). Data from two independent experiments with three to four inserts each (n = 7). Data presented as mean ± SD.

**SI Fig.S8** shows representative EIS data in Bode and Nyquist plot format for epithelial (Caco2/HT29-MTX) and endothelial (HUVEC) barriers following basal incubation with palmitic acid.

**SI Fig. S8.** Palmitic acid treatment: Impedance spectra for the quad-culture showing gel (apical) or membrane (basal) baselines, cell barrier baselines and cell barriers following 24 and 48 hours of palmitic acid treatment in (**A-B**) Bode plot format and (**C-D**) in Nyquist plot format. Plots showing raw data (solid lines) and fitted data (dotted lines) obtained using the resistor (R) and capacitor (C) circuit [R1(R2C2)C1]where R2 is the barrier resistance and metric of interest. (**A** **and** **C**) showing apical epithelial (Caco2/HT29-MTX) barrier changes (0 hrs - R2: 11570 Ω, R2 error: 10.44 %; 24 hrs – R2: 8340 Ω; R2 error: 10.56 %; 48 hrs – R2: 6912 Ω; R2 error: 13.13%) and (**B and D**) showing basal endothelial (HUVEC) barrier changes (0 hrs - R2: 1316 Ω, R2 error: 38.97 %; 24 hrs – R2: 959.1 Ω; R2 error: 37.35 %; 48 hrs – R2: 960.5 Ω; R2 error: 68.88 %).

**SI Table.S1** lists the four individual bacteria as well as the bacteria within the synthetic community, from whose growth supernatants were derived.

**SI Table.S1.** Bacteria grown in mGam media to generate bacterial supernatants

| **Individual bacteria** | **Synthetic community** |
| --- | --- |
| 1. *Segatella copri* (*S. copri*) 2. *Roseburia intestinalis* (*R. intestinalis*) 3. *Parabacteroides merdae* (*P. merdae*) 4. *Bacteroides caccae* (*B. caccae*) | 1. *Agathobacter rectalis* 2. *Akkermansia muciniphila* 3. *Bacteroides fragilis* (nontoxigenic) 4. *Bacteroides uniformis* 5. *Bifidobacterium adolescentis* 6. *Bifidobacterium longum subsp. infantis* 7. *Clostridioides difficile* 8. *Clostridium perfringens* 9. *Clostridium symbiosum* 10. *Eggerthella lenta* 11. *Enterocloster bolteae* 12. *Escherichia coli ED1a* 13. *Escherichia coli IAI1* 14. *Fusobacterium nucleatum subsp. Nucleatum* 15. *Lacrimispora saccharolytica* 16. *Lacticaseibacillus paracasei* 17. *Lactobacillus gasseri* 18. *Odoribacter splanchnicus* 19. *Parabacteroides distasonis* 20. *Parabacteroides merdae* 21. *Phocaeicola vulgatus* 22. *Roseburia intestinalis* 23. *Segatella copri* 24. *Streptococcus parasanguinis* 25. *Streptococcus salivarius* |

**SI Table.S2** provides information relating to the 25 bacteria in the synthetic community, including details relating to phylum, strain, and taxonomy identifier, amongst others.

**SI Table.S2.** Details relating to the 25 bacteria in the synthetic community.

| **Bacteria** | **Phylum** | **Strain** | **TaxID** | **Source** | **Selection criteria** |
| --- | --- | --- | --- | --- | --- |
| *Agathobacter rectalis* | [Bacillota (Firmicutes)](https://www.ncbi.nlm.nih.gov/Taxonomy/Browser/wwwtax.cgi?mode=Undef&id=1239&lvl=3&keep=1&srchmode=1&unlock) | *Agathobacter rectalis, A1-86* | 657318 | DSM 17629 | core gut microbiome |
| *Akkermansia muciniphila* | Verrucomicrobiota | *Akkermansia muciniphila, type strain, Muc* | 349741 | DSM 22959 | probiotic |
| *Bacteroides fragilis (nontoxigenic)* | Bacteroidota | *Bacteroides fragilis nontoxigenic, EN-2, VPI 2553* | 272559 | DSM 2151 | core gut microbiome |
| *Bacteroides uniformis* | Bacteroidota | *Bacteroides uniformis, VPI 0061* | 411479 | DSM 6597 | core gut microbiome |
| *Bifidobacterium adolescentis* | Actinomycetota | *Bifidobacterium adolescentis, type strain, E194a (Variant a)* | 367928 | DSM 20083 | probiotic |
| *Bifidobacterium longum subsp. infantis* | Actinomycetota | *Bifidobacterium longum subsp. infantis* | 391904 | DSM 20088 | core gut microbiome |
| *Clostridioides difficile* | [Bacillota (Firmicutes)](https://www.ncbi.nlm.nih.gov/Taxonomy/Browser/wwwtax.cgi?mode=Undef&id=1239&lvl=3&keep=1&srchmode=1&unlock) | *Clostridioides difficile, 630* | 272563 | DSM 27543 | pathogen |
| *Clostridium perfringens* | [Bacillota (Firmicutes)](https://www.ncbi.nlm.nih.gov/Taxonomy/Browser/wwwtax.cgi?mode=Undef&id=1239&lvl=3&keep=1&srchmode=1&unlock) | *Clostridium perfringens* | 195103 | DSM 756 | pathogen |
| *Clostridium symbiosum* | [Bacillota (Firmicutes)](https://www.ncbi.nlm.nih.gov/Taxonomy/Browser/wwwtax.cgi?mode=Undef&id=1239&lvl=3&keep=1&srchmode=1&unlock) | *Clostridium symbiosum, WAL-14163* | 742740 | HM-309 (BEI Resources) (HMP ID 9474) | conditional pathogen |
| *Eggerthella lenta* | Actinomycetota | *Eggerthella lenta, type strain, 1899 B, VPI 0255* | 479437 | DSM 2243 | causing abdominal sepsis |
| *Enterocloster bolteae* | [Bacillota (Firmicutes)](https://www.ncbi.nlm.nih.gov/Taxonomy/Browser/wwwtax.cgi?mode=Undef&id=1239&lvl=3&keep=1&srchmode=1&unlock) | *Enterocloster bolteae, type strain, WAL 16351* | 411902 | DSM 15670 | core gut microbiome |
| *Escherichia coli ED1a* | Proteobacteria | *Escherichia coli ED1a* | 585397 | Denamur Lab, INSERM | core gut microbiome |
| *Escherichia coli IAI1* | Proteobacteria | *Escherichia coli IAI1* | 585034 | Denamur Lab, INSERM | core gut microbiome |
| *Fusobacterium nucleatum* | Fusobacteriota | *Fusobacterium nucleatum, type strain, 1612A, VPI 4355* | 190304 | DSM 15643 | associated with colorectal cancer and IBD |
| *Lacrimispora saccharolytica* | [Bacillota (Firmicutes)](https://www.ncbi.nlm.nih.gov/Taxonomy/Browser/wwwtax.cgi?mode=Undef&id=1239&lvl=3&keep=1&srchmode=1&unlock) | *Lacrimispora saccharolytica, type strain, WM1* | 610130 | DSM 2544 | forming separate metabolic clade represented by unusual metabolic reactions |
| *Lacticaseibacillus paracasei* | [Bacillota (Firmicutes)](https://www.ncbi.nlm.nih.gov/Taxonomy/Browser/wwwtax.cgi?mode=Undef&id=1239&lvl=3&keep=1&srchmode=1&unlock) | *Lacticaseibacillus paracasei, LPC-37, ATCC No.: SD5275* | 1597 | Dupont Health and Nutrition | probiotic |
| *Lactobacillus gasseri* | [Bacillota (Firmicutes)](https://www.ncbi.nlm.nih.gov/Taxonomy/Browser/wwwtax.cgi?mode=Undef&id=1239&lvl=3&keep=1&srchmode=1&unlock) | *Lactobacillus gasseri* | 324831 | DSM 20243 | probiotic |
| *Odoribacter splanchnicus* | Bacteroidota | *Odoribacter splanchnicus, type strain, 1651/6* | 709991 | DSM 20712 | >= 10^-2 rel. abundance (in at least one sample) and >= 10% prevalence |
| *Parabacteroides distasonis* | Bacteroidota | *Parabacteroides distasonis, ATCC 8503, CCUG 4941, JCM 5825, NCTC 11152* | 435591 | DSM 20701 | >= 10^-2 rel. abundance (in at least one sample) and >= 10% prevalence |
| *Parabacteroides merdae* | Bacteroidota | *Parabacteroides merdae, VPI T4-1, CIP 104202T, JCM 9497* | 411477 | DSM 19495 | >= 10^-2 rel. abundance (in at least one sample) and >= 10% prevalence |
| *Phocaeicola vulgatus* | Bacteroidota | *Phocaeicola vulgatus, type strain* | 435590 | DSM 1447 | core gut microbiome (Mende D. et al, Nat. Methods (2013)) |
| *Roseburia intestinalis* | [Bacillota (Firmicutes)](https://www.ncbi.nlm.nih.gov/Taxonomy/Browser/wwwtax.cgi?mode=Undef&id=1239&lvl=3&keep=1&srchmode=1&unlock) | *Roseburia intestinalis, L1-82* | 536231 | DSM 14610 | probiotic |
| *Segatella copri* | Bacteroidota | *Segatella copri, type strain, CB7* | 537011 | DSM 18205 | opportunistic pathogen |
| *Streptococcus parasanguinis* | [Bacillota (Firmicutes)](https://www.ncbi.nlm.nih.gov/Taxonomy/Browser/wwwtax.cgi?mode=Undef&id=1239&lvl=3&keep=1&srchmode=1&unlock) | *Streptococcus parasanguinis, type strain* | 760570 | DSM 6778 | >= 10^-2 rel. abundance (in at least one sample) and >= 10% prevalence |
| *Streptococcus salivarius* | [Bacillota (Firmicutes)](https://www.ncbi.nlm.nih.gov/Taxonomy/Browser/wwwtax.cgi?mode=Undef&id=1239&lvl=3&keep=1&srchmode=1&unlock) | *Streptococcus salivarius, type strain, 275* | 1304 | DSM 20560 | core gut microbiome (Mende D. et al, Nat. Methods (2013)) |

**SI Fig.S9** provides information relating to the 25 bacteria in the synthetic community, showing how relative abundance of each bacteria changes with passaging.


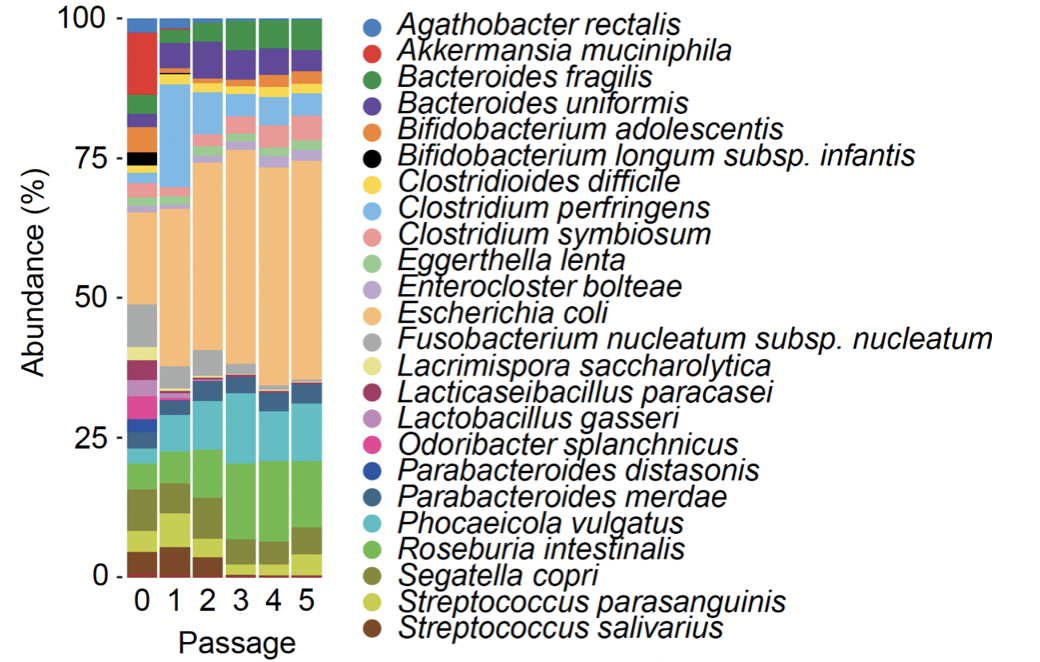


**SI Fig.S9.** Relative abundance of the bacteria in the synthetic community over different passages. Passage four was utilised in this work.

**SI Fig.S10** show the same data as Fig.4A however here separated by compound for clarity.

**SI Fig.S10.** Bacterial supernatant SCFA profiles. Concentration profiles showing relative levels of four SCFAs in the supernatants from individual bacteria and the synthetic community compared to mGam media. Concentrations of **B.** butyric, **C.** isobutyric, **D.** propionic and **E.** isovaleric acid in the supernatants from individual bacteria and the synthetic community compared to mGam media (n=3). Analysed by one-way ANOVA, followed by Tukey’s post-hoc test (n.s. p>0.05; *p<0.05; **p<0.01; ***p<0.001). Data presented as mean ± SD.

**SI Fig.S11** shows representative EIS data in Bode and Nyquist plot format for epithelial (Caco2/HT29-MTX) and endothelial (HUVEC) barriers following apical incubation with supernatant from growth of the synthetic community.

**SI Fig.S11.** Synthetic community treatment: Impedance spectra for the quad-culture showing gel (apical) or membrane (basal) baselines, cell barrier baselines and cell barriers following 24 and 48 hours of synthetic community supernatant treatment in (**A-B**) Bode plot format and (**C-D**) in Nyquist plot format. Plots showing raw data (solid lines) and fitted data (dotted lines) obtained using the resistor (R) and capacitor (C) circuit [R1(R2C2)C1] where R2 is the barrier resistance and metric of interest. (**A** **and** **C**) Showing apical epithelial (Caco2/HT29-MTX) barrier changes (0 hrs - R2: 10620 Ω, R2 error: 9.01 %; 24 hrs – R2: 16560 Ω; R2 error: 11.16 %; 48 hrs – R2: 19840 Ω; R2 error: 10.98 %) and (**B and D**) showing basal endothelial barrier changes (0 hrs - R2: 1137 Ω, R2 error: 21.61 %; 24 hrs – R2: 831.9 Ω; R2 error: 23.47 %; 48 hrs – R2: 825.9 Ω; R2 error: 20.46 %).

**SI Fig.S12** shows confocal immunofluorescence images of the epithelial (Caco2/HT29-MTX) and endothelial (HUVEC) barriers following apical incubation with mGam or supernatant from growth of the synthetic community.

**SI Fig.S12.** Confocal images of the Caco2/HT29-MTX cells with **A.** mGam only and **B.** synthetic community (cells are stained for nuclei (Hoechst; blue) and ZO-1 (red/green)). HUVEC cells with **C.** mGam only and **D.** synthetic community (cells are stained for nuclei (Hoechst; blue) and VE-cadherin (green)). Scale bar: 50 $\mu$m.


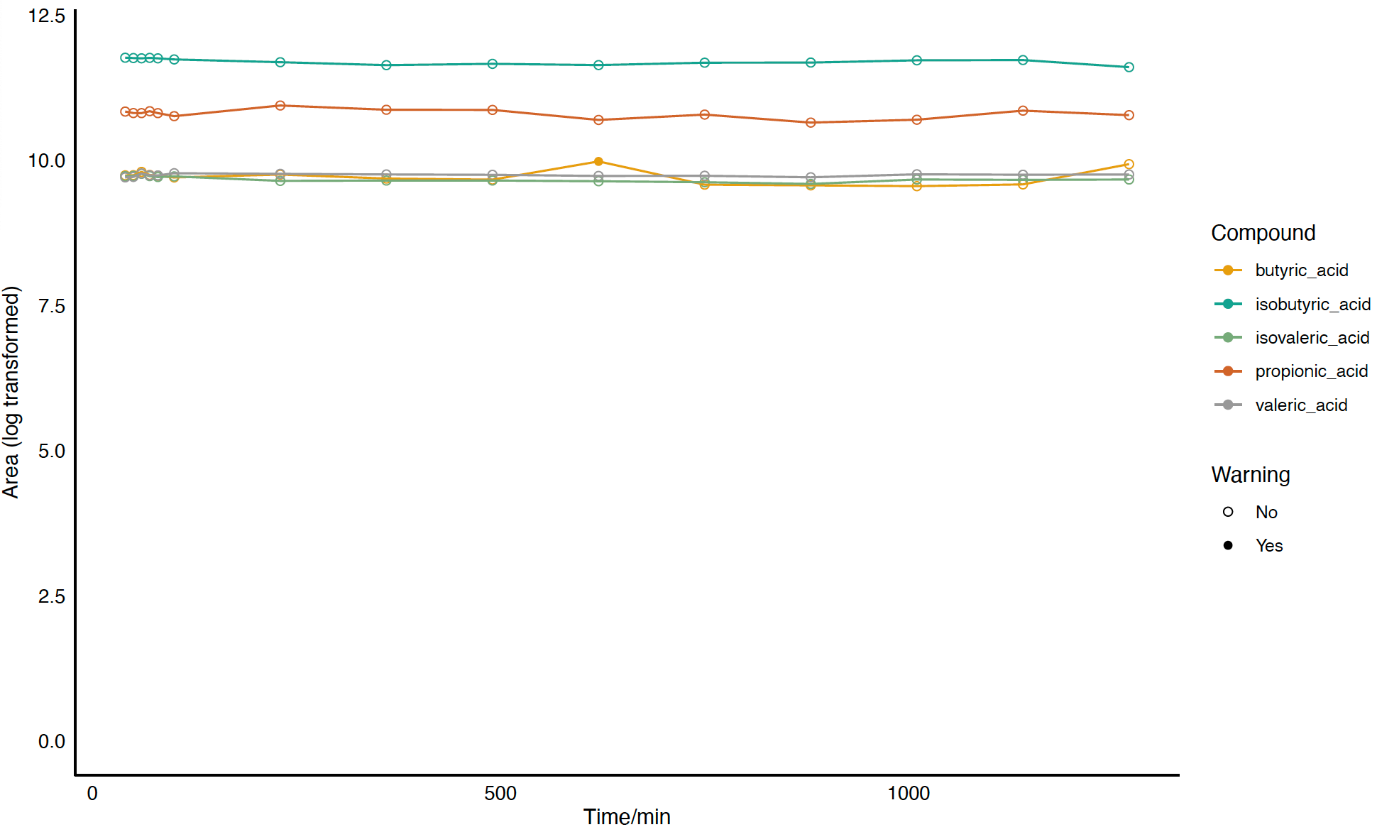


**SI Fig.S13.** Short chain fatty acids were reproducibly detected over the course of the LCMS/MS analysis. Aliquots from each sample were pooled into one sample. This pooled sample was injected regularly over the course of the analysis. Each circle in the plot represents the measured area of the relevant SCFA at the injection of the pooled sample. Filled circles indicate where the measured area of the SCFA varied from the mean by more than 25% of the mean. Overall, the measured area for each SCFA remained stable over the 23-hour run time, demonstrating that the SCFA were stable under the conditions of the run.

**References**

[1] B. E. Dewi, T. Takasaki, I. Kurane, *J. Virol. Methods* **2004**, *121*, 171.

[2] N. J. Machesky, J. M. Rusnak, E. H. Moore, C. B. Dorsey, L. A. Ward, *Toxicon* **2019**, *167*, 152.

[3] P. Hoffmann, M. Burmester, M. Langeheine, R. Brehm, M. T. Empl, B. Seeger, G. Breves, *PLOS ONE* **2021**, *16*, e0257824.

[4] V. M. D. Souza, H. G. Shertzer, A. G. Menon, G. M. Pauletti, *AAPS PharmSci* **2003**, *5*, 17.

[5] L. Peng, Z. He, W. Chen, I. R. Holzman, J. Lin, *Pediatr. Res.* **2007**, *61*, 37.

[6] F. Ouyang, B. Li, Y. Wang, L. Xu, D. Li, F. Li, D. Sun-Waterhouse, *Metabolites* **2022**, *12*, 1028.

[7] M. Gori, A. Altomare, S. Cocca, E. Solida, M. Ribolsi, S. Carotti, A. Rainer, M. Francesconi, S. Morini, M. Cicala, M. Pier Luca Guarino, *Antioxidants* **2020**, *9*, 417.
